# The influence of personality disorder in predicting suicidal behaviour in common mental disorders: A 30‐year study

**DOI:** 10.1002/pmh.1543

**Published:** 2022-04-01

**Authors:** Peter Tyrer, Helen Tyrer, Min Yang

**Affiliations:** ^1^ Division of Psychiatry Imperial College London UK; ^2^ Lincolnshire Partnership NHS Foundation Trust Lincoln UK; ^3^ West China School of Public Health Sichuan University Chengdu China; ^4^ Faculty of Health, Art and Design Swinburne University of Technology Melbourne Australia

## Abstract

Two hundred ten patients with anxiety and depressive disorders were followed up over 30 years. Personality status was assessed at baseline using the Personality Assessment Schedule (PAS), an instrument that classifies personality disorder in a similar way to the new ICD‐11 classification. Assessments of suicidal behaviour were made at 5, 12 and 30 years and suicidal thoughts at 12 and 30 years and analysed by personality status, clinical diagnosis and scores on the General Neurotic Syndrome Scale, a combined diagnosis of mixed anxiety depression and personality dysfunction. Suicide attempts were most frequent in the first 5 years of the study and reduced over time. Baseline personality status was the best predictor of suicide attempts at 5 years (no personality disorder 29.3%, personality disorder 51.6%, *p* = 0.006), and at 12 years (no personality disorder 11.9%, personality disorder 25.7%, *p* = 0.042), but no important differences were found at 30 years, when comorbid mental state disorder was the strongest predictor (*p* < 0.001). Similar but less marked findings were found for the general neurotic syndrome. It is concluded that the presence of personality disorder is a robust predictor of suicidal behaviour in the shorter term but in the long‐term comorbid pathology is a better predictor.

## INTRODUCTION

Suicide accounts for 1.5% of all deaths per year (Fazel & Runeson, 2020). Despite great investment in research and its great importance to public health, there remains no simple clinical intervention that can reliably reduce the incidence of suicide or suicidal behaviour. Many factors, covering socio‐economic, clinical, spiritual, idiosyncratic personal and mental state changes, are relevant to suicidal behaviour (Harris & Barraclough, 1997; Hawton et al., 2003; Lawrence et al., 2016; Spears et al., 2017). Personality status has also been recognized to be important, but almost all the data relate to one condition, borderline personality disorder (Black et al., 2004; Leichsenring et al., 2011).

In the course of planning a long‐term study on the outcome of common mental disorders, we carried out assessments of personality status at baseline and recorded details of suicidal behaviour at 5, 12 and 30 years of follow‐up. The main hypothesis tested with regard to suicidal behaviour was that personality status would be a better predictor of such behaviour than mental illness diagnosis and that this would persist over the longer term.

### Method

The Nottingham Study of Neurotic Disorder was initiated in 1982. Between 1983 and 1987, two hundred ten patients were recruited from general practice psychiatric clinics, which were popular in the United Kingdom at the time (Strathdee & Williams, 1984; Tyrer, 1984). All the patients were seen in Nottingham and initially involved in a randomized trial of treatments (Tyrer, Murphy, et al., 1988). The eligible patients had a DSM‐III diagnosis of either generalized anxiety disorder (GAD), panic disorder or dysthymic disorder (or any mixture of these), determined by the administration of the Structured Clinical Interview for DSM‐III (Spitzer & Williams, 1983), were on no active psychiatric treatment at entry, had no history of schizophrenia, bipolar disorder or alcohol or drug addiction, and gave informed consent.

Personality assessment was made at baseline using the Personality Assessment Schedule (PAS) (Tyrer et al., 1979; Tyrer & Alexander, 1979). The PAS is an interview schedule carried out by a trained observer. It takes about 45–60 min to complete and assesses 24 personality attributes, each on an 8‐point scale. The scores are subsequently classified into two groups, one to assess severity, and the other to the type of personality disturbance, and this predates the adoption of a similar model in the ICD‐11 classification of personality disorder (Tyrer et al., 2019). The PAS is a combined categorical and dimensional scale similar to the Schedule for Normal and Abnormal Personality (SNAP) (Clark et al., 2014). The assessments were made by previously trained independent researchers (all psychiatrists) who had to achieve good agreement with standard vignettes so when assessed together agreement of at least 0.65 kappa was reached using the PAS (Tyrer et al., 1990).

The categorical diagnoses were grouped into four on the basis of a previous factor and principal components analysis. At that time (1979), the categories in the PAS were listed as antisocial, dependent (but subsequently passive‐dependent), inhibited (later anankastic) and withdrawn (later schizoid) (Tyrer et al., 1990). The categorical groups of personality from the DSM‐III classification were also recorded at baseline using a separate algorithm derived from the individual items of the PAS (Tyrer, Alexander, & Ferguson, 1988, pp. 166–167). The same algorithm was used to assess DSM‐III personality disorders when personality status was assessed at 12 and 30 years of follow‐up.

#### General neurotic syndrome and personality status

The three DSM diagnoses covering eligibility for the study were formerly classified as neurotic disorders before the introduction of DSM‐III (American Psychiatric Association, 1980). This notion of neurosis is inextricably associated with the personality characteristics of nervousness, tendency to mood swings with recurrent depressive features, lack of self‐confidence and self‐esteem, a combination of pessimism and reluctance to take risks, and a tendency to engage with others in a dependent role. Such personality features are now included under the term ‘neuroticism’. Because this association is so common it was formally named the ‘general neurotic syndrome’ (Andrews et al., 1990; Tyrer, 1985; Tyrer et al., 1992). This is defined as a combination of mixed anxiety and depressive symptomatology renamed as cothymia (Tyrer, 2001; Tyrer et al., 2003), with dependent and obsessional personality features, and with a history of a first degree relative having similar symptoms (Tyrer et al., 2016). It is one of a proposed group of Galenic syndromes—closely entwined personality and mental state diagnoses (Tyrer et al., 2022)—that is becoming important in practice. In the Nottingham Study the general neurotic syndrome was also postulated to have a negative impact on the outcome of anxiety and depressive disorders.

#### Comorbid mental pathology

The primary outcome at 12 and 30 years was the presence or absence of a DSM disorder apart from minor ones like adjustment disorders (Tyrer, Tyrer, Johnson, & Yang, 2021). By examining suicidal behaviour separated by DSM status, it was possible to determine the influence of comorbid mental state aspects of this behaviour.

#### Statistical analysis

Personality disorder was defined as any condition satisfying the ICD‐11 criteria for the condition (Tyrer et al., 2019). Conversion from the original PAS data had been carried out earlier (Tyrer et al., 2014). Three levels of status of general neurotic syndrome (GNS) were defined by GNS score at <4 (no syndrome), 4–5 (possible syndrome) and 6 and above (definite syndrome). The differences in suicidal behaviour separated by baseline personality disorder and GNS status, of mental state disorder at follow‐up (DSM present or absent), and individual baseline DSM personality disorder were presented in percentages and tested using Fisher's exact test at the 0–5, 6–12 and 13–30 year periods, respectively. Two level logistic models for repeated measures with patients at level 2 and time at level 1, were used to estimate joint impacts of personality status on suicidal behaviours and thoughts over time. There were three time periods for suicidal attempts, and two time periods for suicidal thoughts and admission. In the analysis, personality status, GNS level, DSM status, time period indicators, age and sex were all entered in the same model, which further estimate partial effects of PD, GNS, DSM conditions and change of suicidal behaviours over time by odds ratios with adjustment for age and sex of patients. In another word, independent effects of PD, GNS and DSM status and change over time were estimated by the model.

IBM SPSS v19 and MLwiN 2.3 were used for statistical analysis when required.

## RECORDING OF SUICIDAL BEHAVIOUR

After completion of the randomized trial those participants who agreed to be followed up had three further assessments in which suicidal behaviour was examined. Their clinical notes in both primary and secondary psychiatric care examined at 5 years, they were interviewed in face‐to‐face meetings at 12 years and their primary care notes examined, and at 30 years they were interviewed at a face‐to‐face meeting only. Almost all assessments were made by HT with occasional back‐up from PT. Personality diagnosis was determined by analysis using the original algorithm; its results were not known to the researchers.

Frequency of suicidal thoughts was recorded by asking the question; ‘have you had any thoughts about suicide—of ending your life—recently?’ The answers were recorded.

### Results

Seventy‐one of the patients had died by the time of the 30 year follow‐up. Four of these died by suicide or suspected suicide (open verdict), three with an initial diagnosis of dysthymic disorder and one with generalized anxiety disorder. Two of them committed suicide within 3 years of randomization. A separate analysis showed no significant differences between those with personality disturbance at baseline and those without (Tyrer, Tyrer, & Yang, 2021). The missing data at 30 years were studied carefully in connection with data at other time points and it was concluded that it was appropriate to regard them as ‘missing at random’ (Tyrer, Tyrer, Johnson, & Yang, 2021).

**FIGURE 1:**
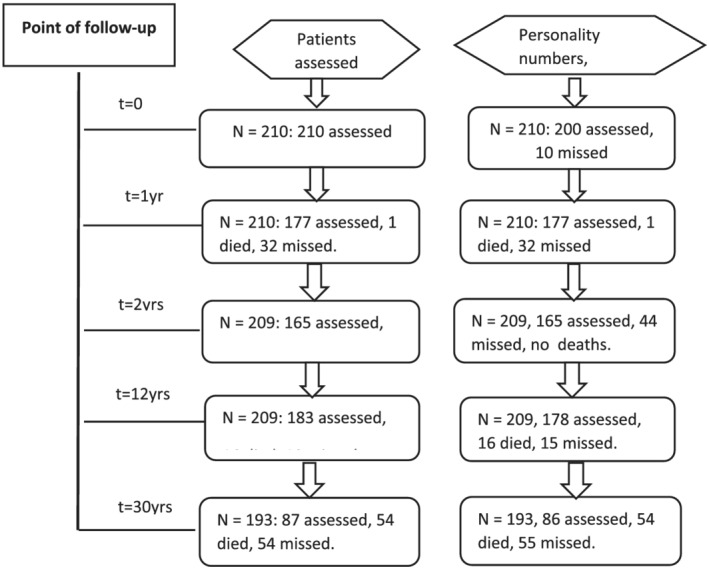
Numbers of patients seen over the 30 year follow up

The follow‐up rates are shown in Figure 1. The details of suicidal thinking and behaviour over the three periods of follow‐up, 5, 12 and 30 years separated by status on the general neurotic syndrome and personality status at baseline are shown in Tables 1 and 2. Suicide attempts were significantly higher in those with the general neurotic syndrome (*p* = 0.029) in the first 5 year period, but not actions, at the 12 year follow‐up, with no important differences at 30 years. In those with personality disorder more suicidal behaviour was shown after 5 years (55.7%) than in those without personality disorder (34.9%) (*p* = 0.006) (Table 2). This was true to a lesser extent at 12 years (*p* = 0.025), and after 12 years and 30 years suicidal thoughts (*p* = 0.018), but not behaviour, were significantly greater in those diagnosed with personality disorder at baseline.

**TABLE 1:** Suicidal behaviour 0–5, 6–12 and 13–30 years follow‐up by baseline general neurotic syndrome status

Suicide measures	GNS < 4	GNS 4–5	GNS ≥ 6	*p* value^a^
	*N* (%)	*N* (%)	*N* (%)	
**0–5 years**				
Suicidal attempts (*n* = 201)				
None	63 (63.6)	21 (67.7)	32 (45.1)	**0.029**
Once or more	36 (36.4)	10 (32.3)	39 (54.9)	
**6–12 years**				
Suicidal attempts (*n* = 189)				
None	80 (86.0)	25 (80.6)	52 (80.0)	0.568
Once or more	13 (14.0)	6 (19.4)	13 (20.0)	
Suicidal thoughts (*n* = 184)				
None	63 (70.0)	19 (65.5)	34 (52.3)	0.079
Occasional to often	27 (30.0)	10 (34.5)	31 (47.7)	
Suicide admission (*n* = 192)				
None	86 (90.5)	29 (93.5)	60 (90.9)	1.000
Once or more	9 (9.5)	2 (6.5)	6 (9.1)	
**13‐30 years**				
Suicidal attempts (*n* = 84)				
None	33 (84.6)	15 (88.2)	18 (64.3)	0.088
Once or more	6 (15.4)	2 (11.8)	10 (35.7)	
Suicidal thoughts (*n* = 88)				
None	32 (76.2)	15 (83.3)	15 (53.6)	0.057
Occasional to often	10 (23.8)	3 (16.7)	13 (46.4)	
Suicide admission (*n* = 84)				
None	36 (92.3)	16 (94.1)	24 (85.7)	0.620
Once or more	3 (7.7)	1 (5.9)	4 (14.3)	

*Note*: A GNS score of 4 or 5 indicates that the general neurotic syndrome is likely; one of 6 or more indicates it is definite (Tyrer, 1989; Tyrer et al., 2016). Items in bold type indicate statistical significance.

^a^ Fishers exact test was used to provide the *p* values.

**TABLE 2:** Suicidal behaviour by baseline personality dysfunction

	No PD	PD	*p* value^a^
	*N* (%)	*N* (%)	
**0–5 years**			
Suicidal attempts (*n* = 196)			
None	82 (65.1)	31 (44.3)	**0.006**
Once or more	44 (34.9)	39 (55.7)	
**6–12 years**			
Suicidal attempts (*n* = 184)			
None	103 (88.0)	50 (74.6)	**0.025**
Once or more	14 (12.0)	17 (25.4)	
Suicidal thoughts (*n* = 179)			
None	78 (69.6)	35 (52.2)	**0.025**
Occasional to often	34 (30.4)	32 (47.8)	
Suicide admission (*n* = 187)			
None	113 (94.2)	57 (85.1)	0.060
Once or more	7 (5.8)	10 (14.9)	
**13–30 years**			
Suicidal attempts (*n* = 83)			
None	37 (82.2)	28 (73.7)	0.426
Once or more	8 (17.8)	10 (26.3)	
Suicidal thoughts (*n* = 87)			
None	41 (80.4)	20 (55.6)	**0.018**
Occasional to often	10 (19.6)	16 (44.4)	
Suicide admission (*n* = 83)			
None	42 (93.3)	33 (86.8)	0.460
Once or more	3 (6.7)	5 (13.2)	

*Note*: PD = personality disorder according to the ICD‐11 classification derived from original classification with the Personality Assessment Schedule (PAS) (Tyrer et al., 2014). Items in bold type are statistically significant.

^a^ Fishers exact test was used to provide the *p* values.

Baseline DSM‐III personality status showed suicidal attempts to be most prominent in paranoid and avoidant personalities in the first 12‐year period. Those with borderline personality disorder had a marginally significant increase in suicidal events in the first 5 years (*p* = 0.064), increasing to a significant increase at 12 years (*p* = 0.003) but not at 30 years. More suicidal thoughts were present among those with paranoid, avoidant and borderline disorders than others. After 12 years, all baseline personality disorders had no impact on suicidal behaviour except for dependent personality disorder which showed more suicide thoughts than those without (*p* = 0.05). This group had a a high proportion of suicide attempts during the first 5 years (*p* = 0.005) (Table 3).

**TABLE 3:** Suicidal behaviour recorded by baseline DSM IV personality disorders

Baseline		Any suicidal attempt		Any suicidal thought		Any suicide admission	
PD	Status	*N* (%)	*p* ^a^	*N* (%)	*p* ^a^	*N* (%)	*p* ^a^
**0–5 years**							
Paranoid	Absence	68 (39.5)	**0.046**				
	Presence	15 (62.5)					
Schizotypal	Absence	79 (41.4)	0.165				
	Presence	4 (80.0)					
Schizoid	Absence	78 (41.5)	0.287				
	Presence	5 (62.5)					
Histrionic	Absence	68 (40.2)	0.147				
	Presence	15 (55.6)					
Antisocial	Absence	71 (0.6)	0.166				
	Presence	2 (57.1)					
Borderline	Absence	70 (40.0)	0.064				
	Presence	13 (61.9)					
Avoidant	Absence	69 (39.4)	**0.020**				
	Presence	14 (66.7)					
Dependent	Absence	68 (38.9)	**0.005**				
	Presence	15 (74.1)					
Obsessive compulsive	Absence	72 (0.0)	**0.034**				
	Presence	11 (68.8)					
Narcissistic	Absence	75 (41.0)	0.160				
	Presence	8 (61.5)					
**6–12 years**							
Paranoid	Absence	25 (14.5)	**0.037**	52 (33.5)	**0.024**	13 (8.0)	0.242
	Presence	8 (33.3)		14 (58.3)		4 (16.7)	
Schizotypal	Absence	31 (16.2)	0.198	62 (35.6)	0.062	16 (8.8)	0.382
	Presence	2 (40.0)		4 (80.0)		1 (20.0)	
Schizoid	Absence	31 (16.5)	0.624	61 (35.7)	0.147	17 (9.5)	1.000
	Presence	2 (25.0)		5 (62.5)		0 (0)	
Histrionic	Absence	26 (15.4)	0.175	55 (35.9)	0.661	12 (7.5)	0.066
	Presence	7 (25.9)		11 (42.3)		5 (19.2)	
Antisocial	Absence	25 (14.3)	**0.011**	56 (34.5)	0.337	13 (7.8)	0.105
	Presence	8 (38.1)		10 (47.6)		4 (19.0)	
Borderline	Absence	24 (13.7)	**0.003**	53 (33.5)	**0.016**	13 (7.8)	0.105
	Presence	9 (42.9)		13 (61.9)		4 (19.0)	
Avoidant	Absence	19 (13.5)	**0.023**	54 (34.0)	**0.028**	14 (8.4)	0.400
	Presence	13 (28.9)		12 (60.0)		3 (15.0)	
Dependent	Absence	29 (16.6)	0.760	56 (34.8)	0.120	15 (8.9)	0.685
	Presence	4 (19.0)		10 (55.6)		2 (10.5)	
Obsessive–compulsive	Absence	29 (16.1)	0.482	61 (35.5)	0.103	15 (8.7)	0.631
	Presence	4 (25.0)		7 (63.6)		2 (13.3)	
Narcissistic	Absence	27 (1.8)	**0.010**	60 (36.1)	0.554	14 (8.0)	0.101
	Presence	6 (46.2)		6 (46.2)		3 (23.1)	
**13–30 years**							
Paranoid	Absence	14 (20.3)	0.491	18 (26.1)	0.109	6 (8.7)	0.617
	Presence	4 (28.6)		7 (50.0)		2 (14.3)	
Schizotypal	Absence	18 (22.8)	0.572	24 (30.4)	1.000	8 (10.1)	1.000
	Presence	0 (0)		1 (25.0)		0 (0)	
Schizoid	Absence	18 (23.1)	0.580	24 (30.8)	1.000	8 (10.3)	1.000
	Presence	0 (0)		1 (20.0)		0 (0)	
Histrionic	Absence	13 (18.1)	0.055	19 (26.4)	0.079	6 (8.3)	0.286
	Presence	5 (45.5)		6 (54.5)		2 (18.2)	
Antisocial	Absence	15 (20.8)	0.697	21 (29.2)	0.727	6 (8.3)	0.286
	Presence	3 (27.3)		4 (36.4)		2 (18.2)	
Borderline	Absence	13 (18.6)	0.143	19 (27.1)	0.197	5 (7.1)	0.106
	Presence	5 (38.5)		6 (46.2)		3 (23.1)	
Avoidant	Absence	14 (19.2)	0.212	20 (27.4)	0.159	6 (8.2)	0.246
	Presence	4 (40.0)		5 (50.0)		2 (20.0)	
Dependent	Absence	14 (18.7)	0.063	20 (26.7)	**0.050**	6 (.0)	0.170
	Presence	4 (50.0)		5 (62.5)		2 (25.0)	
Obsessive–compulsive	Absence	18 (23.7)	0.338	22 (28.9)	0.425	0 (0)	1.000
	Presence	0 (0)		3 (42.9)		0 (0)	
Narcissistic	Absence	15 (19.2)	0.066	22 (28.2)	0.158	6 (7.7)	0.071
	Presence	3 (60.0)		3 (60.0)		2 (40.0)	

*Note*: DSM diagnostic status at baseline was recorded using the algorithm derived from the PAS scores (Tyrer, Alexander, & Ferguson, 1988). Items in bold type are statistically significant.

^a^ Fishers exact test was used to provide *p* values.

Those with a DSM diagnosis at 30 years had much higher rates of suicidal admissions than at earlier times (*p* = 0.006); these were a marked contrast to those with baseline personality disorder (Table 4).

**TABLE 4:** Suicidal behaviour in those with a DSM diagnosis at 12 and 30 years follow‐up

	DSM absent	DSM present	*p* value^a^
	*N* (%)	*N* (%)	
**6–12 years**			
Suicidal attempt (*n* = 184)			
None	85 (87.6)	67 (77.0)	0.079
Once or more	12 (12.4)	20 (23.0)	
Suicidal thoughts (*n* = 183)			
None	76 (79.2)	39 (44.8)	**0.000**
Occasional to often	20 (20.8)	48 (55.2)	
Suicide admission (*n* = 184)			
None	91 (93.8)	76 (87.4)	0.201
Yes	6 (6.2)	11 (12.6)	
**13–30 years**			
Suicide attempt (*n* = 85)			
None	36 (83.7)	31 (73.8)	0.299
Once or more	7 (16.3)	11 (26.2)	
Suicidal thoughts (*n* = 89)			
None	37 (80.4)	26 (60.5)	0.061
Occasional to often	9 (19.6)	17 (39.5)	
Suicide admission (*n* = 83)			
None	39 (100.0)	36 (81.8)	**0.006**
Yes	0 (0)	8 (18.2)	

*Note*: Items in bold type are statistically significant.

^a^ Fishers exact test was used to provide *p* values.

After adjusting for age, gender as well as personality measures each other by joint time multivariate logistic regression analysis, patients with personality disorder were 2.69 and 2.89 times more likely to have suicidal attempts and thoughts than those without PD over the follow‐up period. However, the presence of a DSM diagnosis over the longer follow‐up period presented had a markedly higher impact on suicidal behaviour than that of PD after 5 years, with the adjusted odds ratio between 7.08 and 24.6, although overall, suicidal behaviour in patients were significantly reduced over 30 years by 76% to 89% (Table 5).

**TABLE 5:** Suicidal behaviour and change over time from joint time multivariate logistic model analysis

Covariates	Suicidal attempt (over 30 years) (*N* = 501)	Suicidal thought (over 25 years) (*N* = 296)	Suicide admission (over 25 years) (*N* = 296)
	AOR (95% CI)^a^	AOR (95% CI)^a^	AOR (95% CI)^a^
GNS < 4	(ref)	(ref)	(ref)
GNS4–5	0.93 (0.31–2.77)	1.02 (0.28–3.65)	0.40 (0.04–4.18)
GNS ≥ 6	2.26 (0.99–5.20)	2.48 (0.89–6.90)	0.55 (0.10–3.03)
No PD	(ref)	(ref)	(ref)
PD present	**2.89** (1.33–6.29)	**2.69** (1.07–6.76)	3.60 (0.76–16.9)
No DSM	N/A	(ref)	(ref)
DSM present	N/A	**7.08** (3.34–15.0)	**24.6** (8.73–69.5)
Period (0–5 years)	(ref)		
6–12 years	**0.11** (0.07–0.17)	(ref)	(ref)
13–30 years	**0.14** (0.07–0.25)	**0.28** (0.15–0.53)	**0.24** (0.12–0.48)

*Note*: PD present = personality disorder present at baseline. DSM present = a significant DSM diagnosis was present at follow‐up from 12 years onwards. Items in bold type are statistically significant.

^a^ All covariates were analysed jointly with adjusting for age and sex.

## DISCUSSION

Although personality disorder and suicidal behaviour have been linked frequently, this is one of the first studies that has prospectively examined suicidal behaviour in all types of personality disorder over a long period by face to face assessments (except at 5 years). Most other studies have examined data bases of recorded suicide and found that there is still a risk of serious self‐harm and suicide long after an initial suicide event (Björkenstam et al., 2015; De Moore & Robertson, 1996; Jenkins et al., 2002), but few have carried out prospective studies over a long period in which suicidal behaviour has been monitored. One study by Paris and Zweig‐Frank (1997) involving a 27‐year follow‐up also found worse outcomes in those with dysthymia, and this is consistent with our findings with the general neurotic syndrome, where dysthymia is a very prominent feature (Tyrer, Tyrer, Johnson, & Yang, 2021). In the Nottingham Study, patients were recruited with a DSM diagnosis of common mental disorders and none were severely depressed or suicidal at the time of baseline assessment. The findings could therefore be regarded as representative of suicidal behaviour in common mental illness.

Of the many studies reporting suicidal behaviour in those with personality disorder, most have concerned the borderline condition. There is considerable doubt about the usefulness of borderline personality disorder as a diagnosis (Livesley, 2021; Mulder et al., 2020; Tyrer, 2009) and although it is clearly linked to self‐harm this behaviour is an unfortunate consequence of many other personality disorders. The results of our study, and that of Björkenstam et al. (2015), show that most personality disorders are linked to suicidal behaviour.

The finding that the presence of suicidal behaviour lessens over time in those with personality disorder is consistent with other evidence that personality status often changes in the long‐term (Lenzenweger et al., 2004; Yang et al., 2021) and should not be regarded as an ingrained condition. As the study found that other comorbid mental disorders are much more relevant to suicidal thoughts and actions in the 12–30 year period of the study, there might be less attention paid to personality status and more to other comorbid pathology in the prevention of suicide. We need to be reminded of the strong evidence that the now‐established treatments for the borderline group do not in themselves reduce suicide and only have a limited impact on reducing suicidal behaviour in the shorter term (Hawton et al., 2016). All personality disorders should be taken into account in suicide reduction policies.

## CONFLICT OF INTEREST

PT was the Chair of the WHO ICD‐11 Revision Group for the Reclassification of Personality Disorders (2010–2017). Neither of the other authors has any conflicts of interest to declare.

## ETHICS STATEMENT

Ethical approval for this follow‐up study was granted by Northampton Research Ethics Committee (12/EM/0331).

## Data Availability

The study data are available from Peter Tyrer and Min Yang.
